# Development of an eHealth Intervention Including Self-Management for Reducing Sedentary Time in the Transition to Retirement: Participatory Design Study

**DOI:** 10.2196/63567

**Published:** 2025-01-20

**Authors:** Lisa Hultman, Caroline Eklund, Petra von Heideken Wågert, Anne Söderlund, Maria Lindén, Magnus L Elfström

**Affiliations:** 1 Division of Physiotherapy School of Health, Care and Social Welfare Mälardalen University Västerås/Eskilstuna Sweden; 2 Division of Intelligent Future Technologies School of Innovation, Design and Engineering Mälardalen University Västerås/Eskilstuna Sweden; 3 Division of Psychology School of Health, Care and Social Welfare Mälardalen University Västerås/Eskilstuna Sweden

**Keywords:** behavior change intervention, adherence, integrated behavior change model, autonomous motivation, affective determinants

## Abstract

**Background:**

Having a great amount of sedentary time is common among older adults and increases with age. There is a strong need for tools to reduce sedentary time and promote adherence to reduced sedentary time, for which eHealth interventions have the potential to be useful. Interventions for reducing sedentary time in older adults have been found to be more effective when elements of self-management are included. When creating new eHealth interventions, accessibility and effectiveness can be increased by including end users as co-designers in the development process.

**Objective:**

The aim was to explore the desired features of an eHealth intervention including self-management for reducing sedentary time and promoting adherence to reduced sedentary time in older adults transitioning from working life to retirement. Further, the aim was to develop a digital prototype of such an eHealth intervention.

**Methods:**

The study used the participatory design approach to include end users, researchers, and a web designer as equal partners. Three workshops were conducted with 6 older adults transitioning to retirement, 2 researchers, and 1 web designer. Thematic analysis was used to analyze the data from the workshops.

**Results:**

Participants expressed a desire for an easy-to-use eHealth intervention, which could be accessed from mobile phones, tablets, and computers, and could be individualized to the user. The most important features for reducing sedentary time were those involving finding joyful activities, setting goals, and getting information regarding reduced sedentary time. Participants expressed that the eHealth intervention would need to first provide the user with knowledge regarding sedentary time, then offer features for measuring sedentary time and for setting goals, and lastly provide support in finding joyful activities to perform in order to avoid being sedentary. According to the participants, an eHealth intervention including self-management for reducing sedentary time in older adults in the transition to retirement should be concise, accessible, and enjoyable. A digital prototype of such an eHealth intervention was developed.

**Conclusions:**

The developed eHealth intervention including self-management for reducing sedentary time in older adults transitioning to retirement is intended to facilitate behavior change by encouraging the user to participate in autonomously motivated activities. It uses several behavior change techniques, such as goal setting and action planning through mental contrasting and implementation intention, as well as shaping knowledge. Its active components for reducing sedentary time can be explained using the integrated behavior change model. Further research is needed to evaluate the feasibility and effectiveness of the eHealth intervention.

## Introduction

### Background

Increased sedentary time is common among older adults [1], and it increases the risk of noncommunicable diseases and premature death [2-4]. Sedentary time is the time spent in sedentary behavior for any duration or in any context [5]. Sedentary behavior can be defined as any waking behavior in a seated, reclined, or lying position with an energy expenditure of <1.5 metabolic equivalents (METs) [5]. With an aging population globally [6], there is an urgent need to reduce sedentary time in older adults. This has been found to be possible [7,8] but difficult, as sedentary behavior is complex and multifaceted [9]. In a recent scoping review [8], researchers found that sedentary time can be reduced in community-dwelling older adults using multicomponent interventions. Further, their results showed that information, goal setting, health coaching, self-monitoring, and action planning were included in effective interventions. However, studies reporting effect size reported only a mild to moderate reduction in sedentary time. Consequently, there is a need to develop new interventions with new approaches to reduce sedentary time in older adults.

The transition to retirement could be a favorable time for the introduction of interventions for reducing sedentary time in older adults. This is because retirement leads to changes in health behaviors such as physical activity, sedentary time, and smoking [10,11]. A recent longitudinal cohort study found that 61% of participants made health behavior changes when retiring [12], indicating a window where health behavior interventions, such as interventions for reduced sedentary time, could be timely. Previous research has found that interventions for health behavior change are more effective when elements of self-management are included [8]. Self-management is a person’s own day-to-day management of health promotion or disease, including management of their medicines, emotions, meaningful behaviors, and roles [13].

Previous research has found that older adults often equate sedentary behavior with physical inactivity, and are unaware of the health consequences of prolonged sedentary time, how much time they spend in sedentary behavior, and when they are sedentary [9,14]. Affect has an impact on sedentary time in older adults, as enjoyment can be a motive for taking part in nonsedentary behavior [15] and sedentary behavior [9]. Older adults transitioning to retirement experience sedentary behavior as something related to health and well-being, giving them an opportunity to engage in meaningful activities, such as self-reflection, but also as something unhealthy and related to loneliness and boredom [16].

A recent study found that older adults in the transition to retirement use and desire self-management strategies that influence both affective determinants (related to emotions and affects such as joyfulness) and cognitive determinants (related to cognitive processes and includes attitudes and self-efficacy) for reducing sedentary time [17]. Earlier interventions for reducing sedentary time have primarily targeted cognitive determinants, resulting in positive changes with only a mild to moderate effect size [8]. Phipps et al [18] found that sedentary time is influenced more by explicit affective attitudes than by explicit instrumental attitudes. In recent years, interventions addressing affective determinants have been used in health promotion interventions targeting health behaviors [19,20]. Lithopoulos et al [21] compared affective, instrumental, and self-regulatory information in office workers and found that affective information had the most influence on reducing sedentary time. However, there are no intervention studies targeting both cognitive and affective determinants to reduce sedentary time in older adults transitioning to retirement.

To be effective, interventions must also have the possibility of reaching the intended end users. eHealth, which involves health services and information delivery through the internet and internet-related technologies [22], might offer an opportunity to reach out to large populations in upper middle– and high-income countries, as the rates of internet use and access in these countries are 83% and 93%, respectively [23]. eHealth interventions include but are not limited to mobile device–delivered health interventions (mHealth), text messaging, use of stationary computers and the internet, and telephone calls. A recent systematic review and meta-analysis of the effect of mobile health app interventions on health behaviors in older adults stated that such eHealth interventions can be used to reduce sedentary time, but there is uncertainty regarding the results owing to small sample sizes [24]. Adherence to eHealth interventions is slightly higher than adherence to traditional interventions, which might be due to eHealth interventions being perceived as more fun [25]. Adherence can be further raised through increased acceptability of the intervention by involving older adults in the development process using participatory research design [26]. Involving end users in the development of eHealth interventions is a common approach [27,28] and can increase the quality of the knowledge created, effectiveness of the intervention developed [28], and accessibility among those who are unaccustomed to eHealth [29].

### Aim

The aim was to explore desired features of an eHealth intervention including self-management for reducing sedentary time and promoting adherence to reduced sedentary time in older adults transitioning from working life to retirement. Further, the aim was to develop a digital prototype of such an eHealth intervention.

## Methods

### Design

The study applied participatory design (PD) [26] to allow for the knowledge of older adults transitioning to retirement, researchers, and a web designer to be utilized and valued equally in the eHealth intervention’s development. PD is a research method in which end users are involved as equal partners in the design process [26] and has previously been used to develop eHealth interventions targeting various health behaviors [30-32]. This study is part of a PD project aimed at health promotion through reduced sedentary time in the transition to retirement. The PD project consists of the following 4 phases: need assessment, idea generation, testing and retesting, and evaluation. This study involves the second phase, idea generation, in which needs and opinions are evaluated in order to generate ideas and develop a digital prototype [26].

### Participants

Participants, who were older adults transitioning to retirement, were recruited by the first author (LH) at theme days and meetings held by retirement organizations and municipalities, and through an interview article in the local newspaper. The criteria for inclusion were as follows: age 60-75 years, ability to speak and understand Swedish, ability to read and comprehend the study instructions, and plan to retire within a maximum of 3 years or having been retired for a maximum of 5 years. The exclusion criteria were as follows: presence of a serious disease, severe loss of vision, and severe loss of communicative ability. All recruited participants were newly retired, and none were still working. For further background information regarding the participants, see Tables 1 and 2.

**Table 1:** Participant (older adult) demographics.

Participant	Age, years	Sex	Time retired, years	Mostly sedentary or nonsedentary at the current or last job	Living with other adults	Type of housing	Self-reported daily sedentary time, hours	Previous use of an eHealth intervention including self-management
Participant 1: Older adult	67	Male	4	Sedentary	Yes	House/townhouse	7-8	No
Participant 2: Older adult	68	Female	3	Nonsedentary	No	Apartment	2	Do not know
Participant 3: Older adult	67	Male	2	Sedentary	No	Apartment	8	No
Participant 4: Older adult	67	Female	2	Nonsedentary	Yes	House/townhouse	5	App-based arthritis treatment
Participant 5: Older adult	70	Female	4	Nonsedentary	No	Apartment	6-8	—^a^
Participant 6: Older adult	64	Female	2	Sedentary	Yes	House/townhouse	5	Activity watch

^a^Not applicable.

**Table 2:** Participant (designer and researcher) demographics.

Participant	Age, years	Sex	Description
Participant 7: Researcher	38	Female	Physiotherapist (PhD in physiotherapy) with a research focus on eHealth and participatory design
Participant 8: Researcher	37	Female	Physiotherapist (master’s degree in clinical medical science) with experience in primary and specialist health care
Participant 9: Web designer	39	Female	Adequate education and several years of experience in web development

A workshop group consisting of 9 participants was convened. The group’s size was selected to be large enough to capture different user perspectives and small enough to allow all participants’ voices to be heard. Of the 9 participants, 6 were older adults in the transition from working life to retirement, 2 were researchers participating in the workshop (LH and CE), and 1 was a web designer (Tables 1 and 2). The workshops were led by an experienced workshop moderator to improve the balance of power among participants. The workshop moderator was a female researcher employed by the same university as the authors.

### Ethical Considerations

Prior to recruitment, the study was reviewed and approved by the Swedish Ethical Review Authority (Dnr 2019-03836). Informed consent was obtained from the participants.

### Workshops and Data Collection

**Figure 1:**
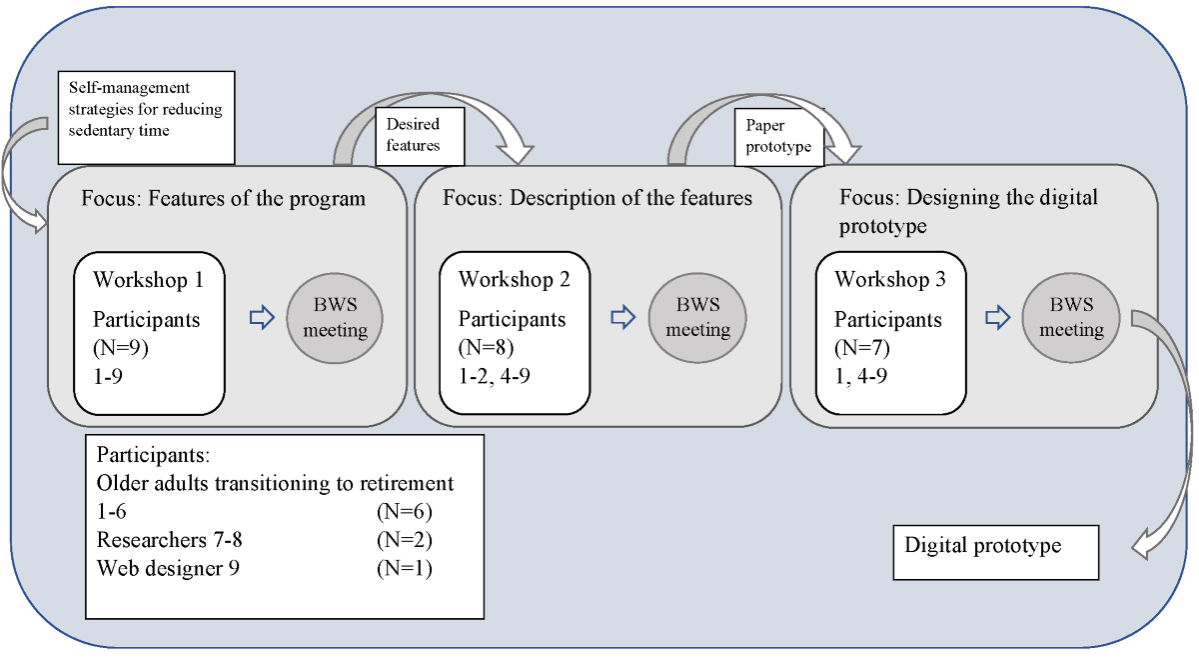
Workshop process map. BWS: between-workshop.

Three workshops were conducted between November 2021 and January 2022 at time intervals of 2 and 8 weeks. All participants were invited to all 3 workshops (see Figure 1 for attendance). The workshops were held in a meeting room at a university. At the beginning of the first workshop, the participants were given written and verbal information about the study, and the study aim and the focus of each workshop were presented (Figure 1), as were the roles of the participants and the moderator. Further the participants were informed that their participation was voluntary and that they could choose to end their participation at any time. Informed consent was signed. The first workshop was planned based on the results of a previous study [17]. In this workshop, focus group interviews were performed with older adults transitioning to retirement, and they involved perceptions of self-management strategies for reducing sedentary time and adhering to the reduced sedentary time. PD tool cards [33] were used at the first workshop, with participants being asked to list on Post-it notes which features in a digital solution they judged to be necessary to operationalize the self-management strategies for reducing sedentary time. This was followed by a discussion. Workshop 2 used 2D mapping [33], taking its starting point in a mind map of features developed from workshop 1. The third workshop used the PD tool user journey [33], with participants working based on a paper prototype developed after workshop 2. Participants were also asked to prioritize the importance of the features, voting for the first, second, and third most important features for reducing sedentary time. The voting was conducted using Post-it notes, with different colored notes used to represent the first, second, and third most important features. After the prioritization of the features, drafts of each feature of the paper prototype were presented, and suggestions for changes were discussed.

After each workshop, the first author (LH) went through the Post-it notes and photographs and listened to audio recordings to create mind maps and outline possible features of the eHealth intervention. Audio recordings were used at this stage to clarify and deepen the understanding of the text written on the Post-it notes. Between-workshop meetings were conducted with the 2 researchers participating in the workshop and 4 additional researchers (co-authors PHW, AS, ML, and MLE) with extensive experience in developing eHealth interventions, behavior change interventions, and gerontologic research. Preliminary results and proposed solutions were presented at the between-workshop meetings, and ideas and questions for the next workshop were discussed. After the last workshop, a digital prototype was developed.

### Data Analysis

After the last workshop, the audio recordings from the workshops were transcribed verbatim. The transcripts were analyzed using inductive thematic analysis [34], with the analysis conducted in 2 steps based on the study aim and the focus of each workshop. The analyses of workshops 1 and 2 focused on desired features and their descriptions (feature analysis). This served as a verification of the paper prototype presented at workshop 3. The analysis of workshop 3 aimed to describe the necessary changes and the participants’ desires regarding the final layout of the digital prototype (change analysis). The first author listened to the audio recordings and read the transcripts to get familiar with the dataset, after which relevant segments were coded at a semantic level. This was in accordance with phases 1 and 2 of the 6 phases of thematic analysis. In phase 3, initial themes were created. These were then checked against the whole data set, and meetings were held with all authors where the themes were discussed (phase 4). The themes were then defined and named (phase 5). Although writing had been carried out since phase 3, the analysis proceeded into the writing of the manuscript (phase 6) as themes were then clarified. The themes from the analysis can be found in Multimedia Appendix 1. The results from the prioritization of the features were summarized and used as a basis for discussion during workshop 3.

## Results

### Overview

The feature analysis resulted in the following 2 themes: general features and features for self-management. The latter consisted of 9 subthemes: finding activities that arouse joy, support in goal setting, information regarding sedentary behavior, measure sedentary time and receive feedback, schedule and planning, rewards, reminders, interact with other users, and timer. These subthemes corresponded to the features of the paper prototype presented at workshop 3. The change analysis resulted in 3 additional themes: evoke positive affect, less is more, and packaging.

**Figure 2:**
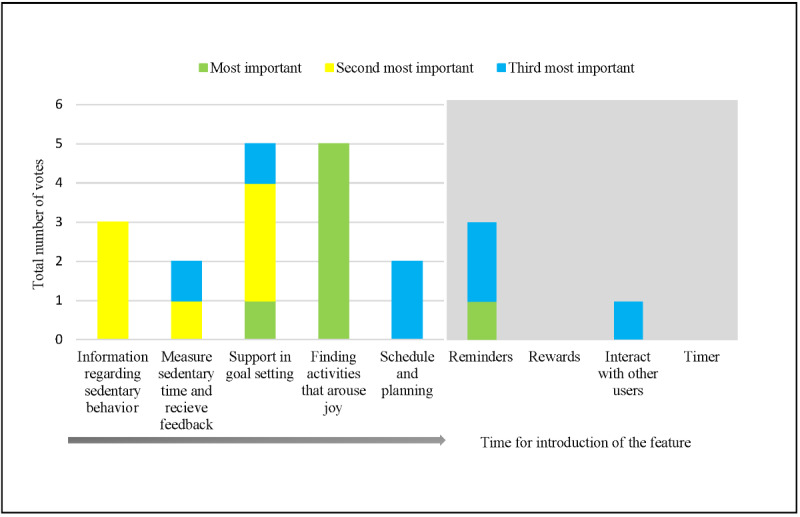
Prioritization of features and time of introduction and inclusion of features in the paper prototype. The gray area represents functions not included in the paper prototype.

The prioritization of the features revealed that the 3 most highly prioritized features in self-management for reducing sedentary time were finding activities that arouse joy, support for goal setting, and information regarding sedentary behavior. More information, such as the order in which participants wanted the features to be presented to the user, can be found in Figure 2.

### Feature Analysis

#### General Features

Participants wanted the eHealth intervention to include basic features, with the possibility to add more features for those who are more technically advanced.

I mean, there are so many people on their way into retirement and there are even more who are retired today who have a very low digitalization level... Yeah, it has to be incredibly simple. Has to be basal. Then you should be able to build on it, maybe, but... The foundation has to be based on something very, very simple.

Participant in workshop 2

To facilitate use, the user should be guided in the order in which the features should be used. To increase accessibility, the eHealth intervention should be available on mobile phones, tablets, and computers. There was concern regarding the inclusion of too many features, as this could lead to increased screen time rather than reduced sedentary time.

The more and more you build into it, it makes it so that, now I have to take a look.

Participant in workshop 1

To further facilitate use, an optional tutorial guiding the user through how the eHealth intervention worked was suggested.

Individualization according to the user’s interests and affections, such as joy, was considered crucial for using the eHealth intervention, reducing sedentary time, and adhering to reduced sedentary time. At the same time, there was concern that individualization would result in the user having to answer too many questions at an early stage, which might exhaust them and prevent their further use of the eHealth intervention.

Yeah, it’s... as soon as you have to go in and start making these kinds of choices that very many people stop.

Participant in workshop 2

#### Features for Self-Management

##### Finding Activities That Arouse Joy

To help them reduce their sedentary time, participants wanted the eHealth intervention to offer suggestions for joyful activities. Since what arouses feelings of joy is individual, participants saw a need to include different types of activities that could be performed in the home, outside the home, by oneself, or together with others, as part of the individualization. They expressed that the eHealth intervention should both allow the user to reflect on what activities they are interested in or could appreciate and provide suggestions for activities based on their areas of interest.

You get to answer questions about what you thought was interesting before and in that way be guided to... where you could look or what more you should do.

Participant in workshop 2

It should also provide information regarding what there is to do nearby when one is newly retired and facilitate contact with retirement organizations through links, information, and a contact person from the organization or activity. Participants expressed a wish for the activities offered by organizations to be presented in the eHealth intervention with their time and location, although they realized the difficulty that gathering this information would entail. Additionally, they wanted the eHealth intervention to include what activities other newly retired people do and enjoy, and provide the user with tips from other newly retired people for fun ways to reduce sedentary time. The difficulty in knowing what activities are available in the local area was expressed by 1 participant as follows:

I think a lot of people have problems with it... How in the world do I find things? What’s here and where [is it], how do I get in contact with groups?

Participant in workshop 1

##### Support in Goal Setting

Participants considered goal setting to be important for reducing sedentary time, but described formulating goals as difficult. They suggested that it could be made easier by offering the user predetermined options or levels of sedentary time as goals, with these goals formulated based on either the maximum time to be spent in sedentary behavior or the number of activities to perform.

Can there be, like: Level 1, Level 2, Level 3, so you don’t have to sit and make it up yourself, sort of?

(You’re thinking that there should be suggestions for goals?)

Yeah, maybe if you’re, you know, completely sedentary. What’s Level 1. Yep. I finished that on the first day. Level 2, and so on.

Participant in workshop 2

Although goal setting was thought to be important for reducing sedentary time, a desire was expressed that this be made optional in the eHealth intervention. Participants expressed that, when formulating a goal regarding reduced sedentary time, they would like to be offered suggestions for what to do instead, and at follow-up, if the goal was not fully achieved, they would like the eHealth intervention to propose adapted suggestions to help them fulfill it in the future.

##### Information Regarding Sedentary Behavior

A need was expressed for information regarding sedentary behavior to be presented to the user at an early stage, so they would know why prolonged sedentary time should be avoided. In addition, participants wanted the information to be offered to the user both as optional short notifications and as static text to be found within the eHealth intervention. They expressed that they would like the information to include the benefits of not engaging in prolonged sedentary time, how long a person can stay sedentary before it is considered unhealthy, and tips on how sedentary time can be reduced. The participants stated that information must be formulated in a positive manner throughout the eHealth intervention. During one of the workshops, participants discussed information regarding sedentary behavior as follows:

That it’s actually stated then that, where’s this limit [for how long you can be sedentary]. That you get that information.

Participant in workshop 2

##### Measure Sedentary Time and Receive Feedback

To set realistic goals, it is necessary to first measure one’s sedentary time. Further, participants saw a need to measure their sedentary time again at goal follow-up. This measurement could be done on specific days rather than daily to reduce the burden on the user. Different ways of taking the measurement were discussed, and while the participants saw the benefit of using sensors in an eHealth intervention, this would require users to always carry their smartphone, which was considered inconvenient. A watch or other device could be connected to the eHealth intervention, but it was thought that this would entail costs that would be unaffordable for some. Participants considered self-reporting one’s sedentary time to be an appropriate solution. Suggested features for self-reporting sedentary time included tapping a stopwatch in the eHealth intervention when one sat down and tapping it again when one stood up, or estimating the time one spent in sedentary behavior. Using a stopwatch was considered unfavorable due to the risk of forgetting or tapping it in the wrong way, and participants did not want to have to carry their phone with them and tap it all day. The estimate of the time one spent in sedentary behavior could be given as an average for a typical day or as an estimate of the current day given in the evening. Participants expressed that after the user had measured their sedentary time, they would need feedback from the eHealth intervention to know whether they had spent too much time in sedentary behavior.

Then you get statistics on how you’ve been sitting. How long you’ve been sitting and how it’s affected your body... Then you learn, okay, now I have to change my habits, if you’re given information.

Participant in workshop 2

##### Schedule and Planning

Participants wanted a schedule or calendar to support their planning and facilitate new routines. It was important for the schedule to be easy to handle, with just a few tabs. It would need to be close at hand. For some, this meant being in their phone, while for others, it meant a printed copy to put on their fridge. Participants said they would like a connection between the proposed activities and the schedule, so that activities could easily be placed in the schedule from the activity menu. For follow-up, the participants wanted the opportunity to check off those activities they had performed as planned.

I think this, that it’s connected to the schedule, is quite good... because if you have to physically enter it in yourself. Then it’s. It’s a form of resistance and an extra step.

Participant in workshop 2

##### Rewards

Participants wanted the eHealth intervention to include rewards at goal fulfillment and as notifications in order to increase their motivation and adherence, and considered it important that the rewards lead to feelings of joy. Which rewards led to joy was said to be highly individual; for instance, some said they would like to have virtual stars and trophies in the eHealth intervention, while others suggested gift cards in line with the user’s interests. To solve the problem of adapting to these differences, participants suggested that the eHealth intervention should urge users to treat themselves with a reward, with the possibility for users to specify in advance what this treat should be. While there was concern that this treat would be something that was unhealthy for the user, it was also discussed that engaging in a fun or amusing activity was rewarding and something to look forward to.

…also has to be something that’s fun that you can look forward to. You can reward yourself.

Participant in workshop 1

##### Reminders

Participants considered reminders to be important for adherence to new routines and said they would like to have reminders about their use of the eHealth intervention, goals, and planned activities. It could be enough to get a reminder in the morning, but as the timing of when reminders are needed is individual, the participants wanted the user to be able to select when the eHealth intervention issued reminders. It was important for the reminders to be phrased positively and give joy, as it was expressed that positively phrased prompts lead to action and adherence more often than other prompts.

Support that’s encouraging is very... We don’t shut it off in the same way as support that’s maybe not encouraging.

That gives [us] a bad conscience instead.

Participant in workshop 1

As what gives joy is also individual, a reminder regarding a planned activity that brings joy could be a way to achieve individualization.

##### Interacting With Other Users

Participants expressed that they would like to have features that provide the opportunity to interact with others to reduce their sedentary time. They wanted the opportunity to share suggestions for activities and tips for reducing sedentary time and to see other users’ suggestions. Moreover, they wanted users to be able to give each other positive feedback to increase their motivation.

...then you’re a group, aren’t you, who sort of ehh can cheer each other on and say good luck and well done and all that.

Participant in workshop 2

A chatroom was discussed as a possible way to facilitate interaction between users. There were concerns regarding the social climate in digital chatrooms. Several suggestions for how to improve it were discussed, and the participants preferred a solution in which users could only comment on each other’s activity suggestions with predetermined answers or positive emojis. This interaction could also serve as an opportunity to initiate meeting up with other users with similar interests, but arranging meetings with unknown users also led to safety concerns.

Then there has to be some kind of block in it so you don’t meet some strange person just to... I was going to say... but I’m just thinking that you can include the security aspect in it.

Participant in workshop 1

##### Timer

Participants wanted a timer with an alarm set to 30 minutes to avoid prolonged sedentary time, until the new behavior had become a habit. It was proposed that the timer could be started when the user sat down or could be set in advance to go off every 30 minutes during a selected period.

I might need ehh a pling, like: whoops. Now it’s been 30 minutes.

Participant in workshop 1

...when you describe how you live. I don’t live half as physically active... I live more “small”. Thirty minutes, fine, and then I leave.

Participant in workshop 2

It was also suggested that, rather than simply sounding an alarm when the timer finished, the eHealth intervention could suggest joyful activities that were prespecified by the user.

### Paper Prototype

At the third workshop, a paper prototype of an eHealth intervention including self-management for reducing sedentary time through joyful activities was presented. The prototype was planned as a website solution with important functions as well as information and links to websites and mobile apps. The prototype was designed to introduce the user to the features in the order chosen by the participants during the workshops. The user was first shown information regarding sedentary behavior, then encountered features for measuring sedentary time, and finally received support for goal setting, the selection of joyful activities, and the planning feature (Figure 2).

Brief paragraphs regarding sedentary behavior were presented, which could be shown to the user when they started using the eHealth intervention and could be found in the eHealth intervention at any time if the user wanted to read all the information.

**Figure 3:**
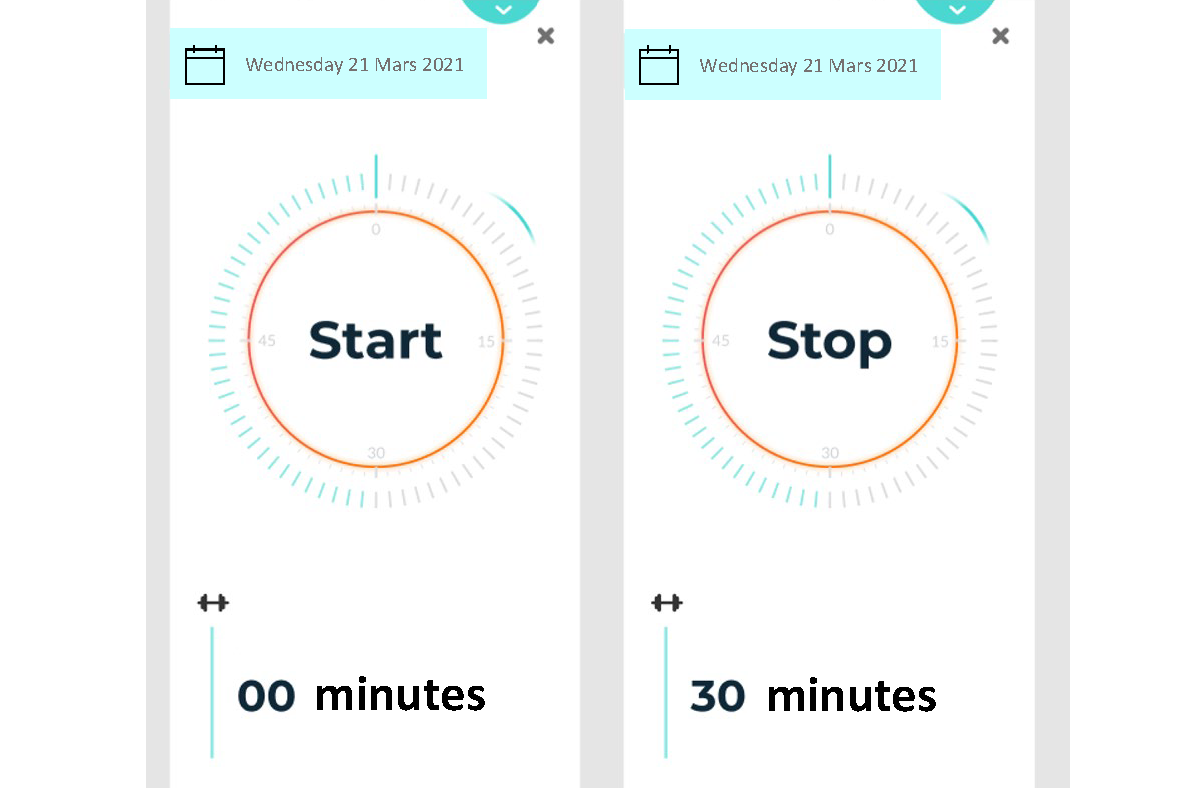
One of the tools presented at workshop 3 for measuring sedentary time.

Two tools for measuring sedentary time were presented. One was a self-assessment tool that asked the user to assess how many hours they had been performing different sedentary behaviors during the day, such as eating, working, or commuting, and the other was a stopwatch for the user to start when they began being sedentary (Figure 3). Alongside the tools for measuring sedentary time, the user would receive adapted feedback based on different time intervals of sedentary time.

The prototype included the design of a support for goal setting. The user was given a written prompt asking them to formulate a goal regarding their sedentary time, and they received support for how this goal should be formulated, with examples of goals shown to them. They were then asked to focus on how they would feel if their sedentary time was reduced and what obstacles they might face, and to make a plan for how to overcome these obstacles. When the goal was then evaluated at follow-up, the user was asked to choose whether it was far from being achieved, nearly achieved, or fully achieved, and then received customized feedback.

**Figure 4:**
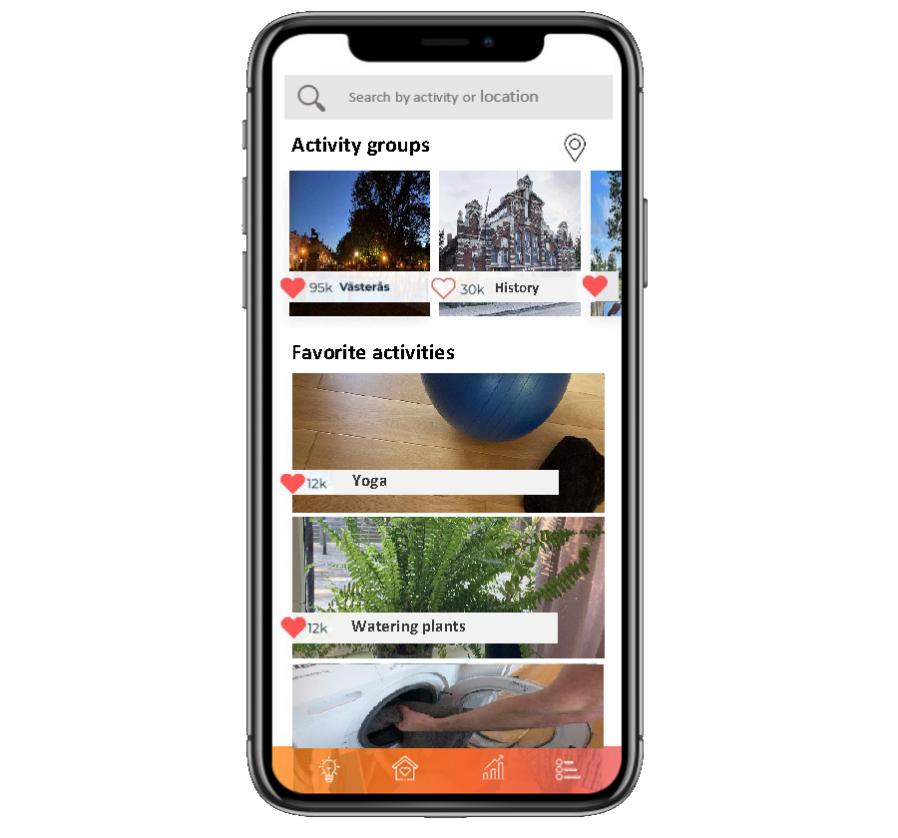
Feature for finding activities that arouse joy from the paper prototype presented at workshop 3.

The feature providing support for finding activities was presented to participants as a scroll list with activities shown along with a brief description and a photo (Figure 4). The scroll list could be sorted based on interests, local areas, and activities performed with others or by oneself. Tips on how to reduce sedentary time were offered as a bulleted list.

The schedule was presented both as a colorful printable schedule designed to evoke positive and cheerful emotions, and as a copy of the schedule from an iPhone and iPad. The user could write their planned activities digitally or manually on the printed schedule. The copy of the schedule from the iPhone and iPad was used to discuss whether the calendar found in a user’s own phone could be used as a schedule.

In the prototype, rewards were delivered at goal evaluation as brief positively formulated phrases adapted to the user’s goal achievement. As the prototype was designed as a website, the possibility to include reminders and interactions with other users was limited. It was suggested that the user make notes regarding planned activities and scheduled follow-ups, preferably in a calendar with the possibility to send notifications. No timer function was included in the prototype; instead, the suggestion to set an alarm when one was to be seated for a prolonged time was included among the tips for reducing sedentary time.

### Change Analysis

#### Evoke Positive Affect

Participants stated that the eHealth intervention’s first impression and introduction should arouse interest to facilitate further use.

If nobody opens this. Then nothing’s worth anything. I mean, there has to be something that captures your interest.

Participant in workshop 3

They suggested that this could be done by having celebrities or cartoons convey the information or by having the user answer questions regarding sedentary time. While the participants considered it important to arouse interest in the eHealth intervention’s introduction, when it came to the delivery of information, they expressed that credibility was important.

Isn’t it two different things we’re talking about... One’s about capturing attention. Come look at our website. [mm] There you could have animated figures, I’m thinking... But when I open the website, then I’d really like to have, to know that there’s evidence behind this in some way, I mean. Ehh am I thinking about it the wrong way?

Participant in workshop 3

They suggested that, to achieve credibility, the information could be presented by a physician or physiotherapist, or by people who were newly retired and could share their experiences. It was stated that all information in the eHealth intervention should be more positively phrased and that when information was evidence-based, this should be clearly stated.

#### Less is More

Overall, the participants wanted the text and information in the paper prototype to be shortened and simplified. The group agreed to choose the tool presented for the self-assessment of sedentary time, as it was considered easier to use than a stopwatch. However, there were concerns regarding whether people who spend a prolonged time in sedentary behavior would take the time to fill in such a form and the fact that it is difficult to make such an assessment. As a solution, it was suggested that the user could fill in an estimate of their total time in sedentary behavior or enter data from an activity watch if they used one. The participants pointed out the need to adapt the assessment form to better suit those who are retired, suggesting that the activities mentioned on the form be replaced with time intervals as every day is different for a person who is newly retired compared to when they had a job. They also suggested that the feedback be changed to give the user a clear overview of when prolonged sedentary behavior becomes unhealthy and whether there are different levels when it comes to harmful amounts of sedentary time, and if so, how close they are to moving on to the next level.

The content of the support for goal setting was considered helpful, but the layout was perceived as cumbersome and too extensive. It was suggested that the text be shortened and reformulated to use more accessible language.

I hadn’t finished reading... There’s too much text.

Participant in workshop 3

The participants pointed out the importance of letting the user know that they do not need to make massive changes to reduce their sedentary time and that small changes and activities in the home could suffice.

It’s also so important that there’s information about that the small things in daily life is enough.

Participant in workshop 3

They suggested that this could be done by sorting activities as “activities in the home” or “start small.” The group was positive about simplifying the eHealth intervention by including links to organizations rather than giving information on each activity offered by the organizations.

The participants considered the rewards offered in the prototype to be sufficient. As the prototype was designed to encourage the user to engage in activities that bring positive affect and emotions, and to remind them of their feelings at goal fulfillment, the participants did not feel further rewards were needed.

The participants mentioned wanting the opportunity to interact with other users within the eHealth intervention. However, at this point, they agreed that the possibility to find activities with others in terms of links and information regarding retirement organizations and local events was sufficient for interacting with others.

Like we’ve talked about before, it was more this thing of being able to interact with other users. I mean, it’s...

not possible now

We’ve dropped that a bit.

But then you can find the organizations, you find them via this.

Participant in workshop 3

To keep the features included in the eHealth intervention to a minimum, a timer was not included; instead, among the tips provided, the user would be given a recommendation to use a timer in other mobile apps or use the timer feature on their phone. The participants discussed the notion that a timer might be useful for those who prefer not to enroll in planned activities with others and would rather perform activities in the home.

At the third workshop, participants did not express a need for a calendar or schedule in the eHealth intervention, as they preferred to write down their planned activities in the calendar on their phones rather than in the calendar suggested at the workshop. They felt that those who do not use the calendar on their phones would probably not use a digital eHealth intervention for planning but likely already use a paper calendar. Therefore, it was suggested that the eHealth intervention should simply suggest that the user write down their planning so that they could choose the type of planning tool they prefer. This was expressed as follows:

I mean, maybe it’s a bit much for those who don’t have it in their phone. I was thinking of this... on the website.

You don’t think they have a calendar on the fridge?

I don’t think they go to their computer and [fill it in].

Participant in workshop 3

#### Packaging

The participants agreed that the eHealth intervention should be delivered as a website rather than a mobile app, as it was important that the intervention be accessible from a computer so it would be easier to operate. However, choosing a website rather than a mobile app limited the possibility to include certain features. For instance, the participants would have liked the eHealth intervention to send reminders to promote adherence, and they suggested that the calendar function on the user’s phone could be used to remind them of planned activities (although they still expressed a desire for the possibility to send a daily reminder or a reminder if the eHealth intervention was not being used as planned). They also wanted to be able to log in to the eHealth intervention in order to save their goals and interests digitally to facilitate the use of the eHealth intervention and for goal follow-up.

Participants expressed a wish for information to be delivered in different ways, allowing the user to listen to or read information, or sometimes get it as a video. It was suggested that, during goal setting, the user should be able to listen to the information, as participants experienced that the spoken word was more appealing and evoked more emotions in this context.

Yeah, I thought about it... that you said it in a much more appealing way than the text does. When you said “Think about what you want to achieve. Imagine how...”, then you get a feeling. I don’t get that here [shows the paper].

Participant in workshop 3

## Discussion

### Principal Findings

To our knowledge, this is the first study to develop a self-management eHealth intervention for reducing sedentary time and promoting adherence to reduced sedentary time, which has been created for and with older adults in the transition to retirement. The study used PD [26] to include end users, researchers, and a web designer as mutual partners in the development process. The study has contributed new knowledge regarding features of this type of eHealth intervention and the timing of these features. The most important features were those involving finding activities that arouse joy, having support in goal setting, and getting information regarding sedentary behavior. The workshop participants regarded the order in which the included features are presented to the user as important: The user first receives knowledge regarding sedentary behavior, then encounters features for measuring sedentary time and for support in setting a goal, and lastly gets support in finding joyful activities to perform in order to avoid being sedentary. The results highlight the need for such an eHealth intervention to be concise, accessible, and enjoyable to suit its end users.

### Comparison With Prior Work

This study differs from previous research [7,8] as it specifically targets older adults in the transition to retirement and involves them as co-designers in the development of an eHealth intervention including self-management for reducing sedentary time and promoting adherence to reduced sedentary time.

Participants perceived it as important for the eHealth intervention including self-management to use positive phrasing to increase both its use and users’ adherence to reduced sedentary time, expressing that positively phrased prompts lead to adherence more than negatively phrased ones. Previous researchers have found positive affect to be connected to the use of eHealth interventions to increase adherence in older adults [25] and positively phrased prompts to be more effective in older adults [35]. Overall, this indicates the importance of an eHealth intervention that evokes positive affect to promote adherence to reduced sedentary time when transitioning to retirement.

The features considered to be the most important for reducing sedentary time in the transition to retirement were those involving finding joyful activities. The importance of joyfulness in reducing sedentary time and increasing physical activity in older adults has been found in previous research [15,36]. In this study, participants stated the importance of receiving suggestions for what to replace sedentary time with, rather than just getting a prompt to break their sedentary behavior. The results further highlight the need for awareness of the diversity in the activities that people consider joyful and for the inclusion of information on simple joyful activities that can be performed in one’s own home to reduce sedentary time.

The eHealth intervention developed in this study includes features for information, measurement, goal setting, and planning, which have been used previously to reduce sedentary time in older adults [7,8] and have been found to be acceptable behavior change techniques for older adults to reduce their sedentary time [9]. The results of this study show that these behavior change techniques are also desired specifically when it comes to an eHealth intervention including self-management for reducing sedentary time, which targets older adults in the transition to retirement.

During the third workshop, self-assessment was chosen as the method for measuring one’s sedentary time. As objectively measured sedentary time is more accurate than self-assessed sedentary time (total sedentary time is often underestimated [37]), the inclusion of objectively measured sedentary time might provide the user with more reliable results. However, doing so would mean adding a device, such as a smartwatch, and this would entail a cost to the user or require them to always carry their phone, which the participants considered inconvenient. The included feature for self-assessment also provides the user with more contextual information, which is an advantage of using self-assessed sedentary time [38].

Among the included features are behavior change techniques that target both affective determinants (eg, affective attitudes) [39] and cognitive determinants (eg, cognitive attitudes and self-efficacy) [40] for reduced sedentary time. The feature for goal setting and planning in the developed eHealth intervention is based on mental contrasting and implementation intention (MCII) [41], which has previously been found to be effective in health behavior change [42]. The inclusion of MCII also responds to the participants’ wish to include goal setting and planning as well as an opportunity to consider activities to replace their sedentary time, which was discussed during the workshops. In the developed eHealth intervention, the user is asked to consider a positive affective outcome, which has been used with success in previous research targeting health behaviors [20]. The inclusion of a feature targeting affective determinants when reducing sedentary time in older adults transitioning to retirement can be an effective approach, as previous research has found that sedentary behavior is largely an affect-driven behavior [18]. Further, Lithopoulos et al [21] found that an intervention targeting affective determinants for reducing sedentary time in the office was effective in the short term.

Previous research has described sedentary behavior as a habitual behavior performed in unawareness [9]. This speaks for the inclusion of features, such as a timer, to increase users’ awareness of when they are in prolonged sedentary behavior and thereby promote their adherence to reduced sedentary time. However, Suorsa et al [43] found that an activity tracker was not sufficient for reducing sedentary time in retirees. In this study, none of the participants rated a timer feature as important, and it was not included in either the paper or the digital prototype. However, among the tips the eHealth intervention offered for reducing one’s sedentary time was the suggestion to set a timer when engaged in sedentary behaviors.

The approach of the eHealth intervention for achieving behavior change, which was developed here, can be understood based on the integrated behavior change (IBC) model for health behavior [44]. The eHealth intervention is designed to guide the user in reducing their sedentary time by finding activities that they are autonomously motivated to perform, meaning that the intention to perform a health behavior is dependent on whether the behavior evokes positive affect and is in line with a person’s interests and true self [45,46]. Further, the developed eHealth intervention includes information, self-monitoring, and feedback on behavior that can influence users’ attitudes and perceived behavioral control regarding the behavior change [40,47]. Lastly, the feature for goal setting includes affective MCII, which can influence behavior through implicit pathways [48,49], as well as elements of action planning, which are included in the IBC model. The use of the IBC model can offer an understanding of how the developed eHealth intervention including self-management is intended to reduce sedentary time and promote adherence to reduced sedentary time, but further studies are needed in order to evaluate the eHealth intervention’s feasibility and effectiveness.

### Limitations and Strengths

Carefully considered choices were made to increase the study’s trustworthiness (credibility, dependability, and transferability) [50]. The study’s methodological approach is a strength, as it enabled the inclusion of older adults in the transition to retirement as co-designers. They are the only ones who hold the tacit knowledge that is needed to develop an eHealth intervention in line with their needs and preferences. The participants also included a web designer and 2 researchers, who contributed knowledge regarding web development and health behavior change. This combination of participants may contribute to the development of an effective and acceptable eHealth intervention as both tacit and explicit knowledge are included in the development process, which strengthens the study’s credibility.

The credibility was further strengthened by a workshop leader guiding the workshops to ensure that everyone had the opportunity to speak and to avoid placing additional power with the researchers. Moreover, the workshops started with an icebreaker, and participants were offered coffee and fruits during the meetings to create a pleasant atmosphere. Three PD tool cards, 2D mapping, and a user journey tool were used to encourage active participation among the participants. These tools were chosen because they have been used successfully in PD research [33], and they ensured that all participants were encouraged and had the possibility to be active during the workshops. The recruitment of participants at theme days and retirement organization meetings might have led to the inclusion of participants with a greater interest in such activities than the population, possibly affecting the study’s credibility and transferability. This was compensated by the recruitment of participants through a local newspaper. Additionally, the participants expressed their differences through participation and interest in activities held by organizations during the workshops. The older adults in the transition to retirement who participated in this study lived in different types of housing and reported different levels of daily sedentary time as well as levels of sedentary time at their latest jobs, which indicates a spread among them and thus increases credibility. The study’s confirmability was strengthened as, during the third workshop, the participants were given the opportunity to discuss and further develop the paper prototype developed from workshops 1 and 2. Further, the feature analysis was used as a verification of the paper prototype to ensure that no important data had been excluded.

### Conclusion

An eHealth intervention including self-management for reducing sedentary time and promoting adherence to reduced sedentary time in older adults transitioning to retirement was developed in a co-design approach involving representatives of end users, a web designer, and researchers. Participants desired features for providing information regarding sedentary behavior, measuring sedentary time, goal setting, finding joyful activities, and making plans and a schedule, and expressed that these should be presented to the user in this order. The most important features for reducing sedentary time were said to be those involving finding joyful activities. Further, participants expressed a desire for such an eHealth intervention to be concise, accessible, and enjoyable in order to be suitable for older adults transitioning to retirement. The developed eHealth intervention strives to support behavior change based on the IBC model by focusing on autonomously motivated activities and affective and cognitive determinants through implicit and explicit pathways, and includes action planning. It will be further tested for feasibility and effectiveness in upcoming studies.

## Appendix

Multimedia Appendix 1 Theme summary.

## Abbreviations

IBC: integrated behavior change
MCII: mental contrasting and implementation intention
PD: participatory design
