# Supplementary material for: Development of an eHealth Intervention Including Self-Management for Reducing Sedentary Time in the Transition to Retirement: Participatory Design Study
Source: JMIR Form Res. 2025 Jan 20;9:e63567. doi: 10.2196/63567 (PMC11791440; doi:10.2196/63567)
Supplement: Multimedia Appendix 1 [file formative_v9i1e63567_app1.docx]

**Multimedia Appendix 1.** Theme summary.

|  | Theme | Subtheme | Characteristics | Example of code |
| --- | --- | --- | --- | --- |
|  |  |  |  |  |
| **Feature analysis** |  |  |  |  |
|  | General features |  | General features of the program which do not refer to self-management | A base which is easy for the inexperienced user |
|  | Features for self-management | Finding activities that arouse joy | Reduce sedentary time through joyful activities | What gives joy is subjective |
|  |  | Support in goalsetting | How it is experienced to set goals and how this can be facilitated by different features | It is difficult to set goals |
|  |  | Information regarding sedentary behavior | What information is needed to reduce sedentary behavior and how the program can provide these features | Information about what is enough |
|  |  | Measure sedentary time and receive feedback | Why and how to measure sedentary time and receive feedback | The disadvantage of a sensor is that you have to carry it with you |
|  |  | Schedule and planning | Making a plan and writing it down | Write down new routines |
|  |  | Rewards | When to get rewards and what these rewards could be | Get a reward at goal achievement |
|  |  | Reminders | To get notifications from the program regarding its existence, goals and activities | Reminders that make you happy |
|  |  | Interacting with other users | Be able to see and contact other users of the program | Share tips and activities |
|  |  | Timer | Get support to not be seated for a longer period of time | Set a timer for 30 minutes |
| **Change analysis** |  |  |  |  |
|  | Evoke positive affect |  | Affect experienced of the user will influence use of the program. Different parts of the program should target different affects | The introduction of the program must arouse interest |
|  | Less is more |  | The request to reduce content and simplify and also that sedentary can be reduced through small changes | Start small |
|  | Packaging |  | How the features should be conveyed for the user | Get the information in a video |
